# A Unique Retinoscopic Reflex Pattern in the Initial Stages of Keratoconus and Its Clinical Significance for Early Keratoconus Detection: A Case Report

**DOI:** 10.7759/cureus.74808

**Published:** 2024-11-30

**Authors:** Ahmed Almaweri

**Affiliations:** 1 Department of Optometry, Noor Alyemen Eye and E.N.T Consulting Center, Sana'a, YEM; 2 Department of Optometry, Faculty of Optometry, Al-Neelain University, Khartoum, SDN

**Keywords:** case report, detection, initial stages, keratoconus, retinoscope

## Abstract

Keratoconus is a bilateral eye anomaly in which the cornea develops gradually, becoming steeper and thinner, causing irregular astigmatism and myopia. This unique case report highlights an atypical retinoscopic reflex that can be observed in the initial stages of keratoconus. While the reflex deviates subtly from the normal form, exhibiting a slightly distorted, irregular, and non-scissoring pattern, it differs significantly from the well-documented "scissor reflex," which is characteristic of moderate to advanced stages. This finding draws attention to the potential utility of the retinoscope in the early detection of keratoconus, particularly in settings with limited access to specialized diagnostic tools. Here, I report a case of a young woman, aged 19 years, who presented with symptoms of glare. During the retinoscopy examination, I observed a distorted and irregular appearance of the retinoscopic reflex, particularly in the left eye, in contrast to that of the right eye. Based on this finding, I suspected the presence of keratoconus. Corneal tomography was used to confirm the diagnosis of keratoconus, and the result was positive for keratoconus. This case report offers a novel clinical presentation that underscores the diagnostic challenges in the initial stages of keratoconus, making it a valuable addition to the literature. The primary lesson learned from this study is that the retinoscope plays a crucial role in the early detection of keratoconus.

## Introduction

Keratoconus is a bilateral eye anomaly in which the cornea develops gradually, becoming steeper and thinner, causing irregular astigmatism and myopia [1]. Most cases of keratoconus appear in the teenage years or early adulthood and stop progressing by the time patients reach their forties [2]. Multiple factors, including environmental, biochemical, biomechanical, and genetic factors, contribute to the progression of keratoconus [3]. Saudi Arabia has the highest reported rate of keratoconus, with almost 4.8% of people between the ages of six and 21 years seeking treatment for non-eye problems at specialized hospitals being diagnosed with keratoconus [4].

Moderate to severe keratoconus is relatively easy to diagnose due to irregular astigmatism and specific classic signs, such as a thin cornea, Vogt's striae, Munson's sign, Fleischer's ring, and Rizzuti's sign. However, detecting mild forms of keratoconus in patients with normal vision and minimal or no manifestations is challenging [5].

The retinoscope is now a fundamental tool for optometrists to determine eye refraction. It is an inexpensive, easily available, portable, and uncomplicated instrument that is highly accurate and trustworthy for identifying keratoconus. Furthermore, the retinoscope is also effective in the initial stages of keratoconus [6]. Keratoconus is an abnormality where the cornea becomes bulging or protrudes, which frequently leads to irregular astigmatism. This irregularity can be seen during retinoscopy as a split retinal reflex, often referred to as a "scissoring reflex" [6].

This case report focuses on how it is possible to manually detect and identify the early stages of keratoconus using a retinoscope. This report also provides a novel description of the retinoscopic reflex pattern observed in the initial stages of the disease, which has not been extensively documented or described in previous studies. This study presents a rare and practical model for the early detection of keratoconus. Early detection of keratoconus is of utmost importance for effective management and monitoring of progression.

## Case presentation

A young female, aged 19 years, presented to our eye center for an eye examination, complaining of glare, and she was not wearing glasses. She has been diagnosed with hypopituitarism and is under the care of an endocrinologist. She is currently receiving hormonal replacement therapy.

Her ocular history was unremarkable, with no history of vernal keratoconjunctivitis (VKC) and no family history of keratoconus. Eye examinations and all interventions were performed in the Ophthalmology and Optometry Department of the Noor Alyemen Eye and E.N.T. Consulting Center in Sana'a, Yemen. These examinations included visual acuity assessment with and without glasses, objective and subjective refraction, slit-lamp evaluation of the eye and adnexa, fundus check-up, and corneal tomography (Pentacam). The visual acuity without correction for the right eye was 20/25, and for the left eye, it was 20/30. The visual acuity with best correction (BCVA) for the right eye was 20/20 with −0.25/−0.50 x 180, and for the left eye, it was 20/20 with −0.25/−1.00 x 180.

During the retinoscopy examination, I observed a distorted, irregular, and non-scissoring retinoscopic reflex in the left eye; the reflex pattern in the right eye was within normal limits. Although this abnormal appearance of the retinoscopic reflex did not perfectly align with the typical reflex pattern seen in keratoconus (the scissoring reflex), I suspected the presence of keratoconus based on these findings. In accordance with these findings from the optometry clinic, the patient was referred to a corneal specialty clinic for further evaluation.

Upon eye examinations, both eyes showed no biomicroscopic signs of keratoconus, and other findings in the eye and adnexa were within normal limits. Additionally, the fundus was normal. Intraocular pressure (I.O.P.) was measured by a handheld rebound tonometer (iCare tonometer) at 10:00 AM, with measurements of 13 mmHg for the right eye and 11 mmHg for the left eye.

Corneal tomography was performed in the cornea clinic to confirm the presence of keratoconus. The result was positive for keratoconus; the right eye showed subclinical keratoconus, while the left eye was at an early stage (Grade 1) of keratoconus.

**Figure 1:**
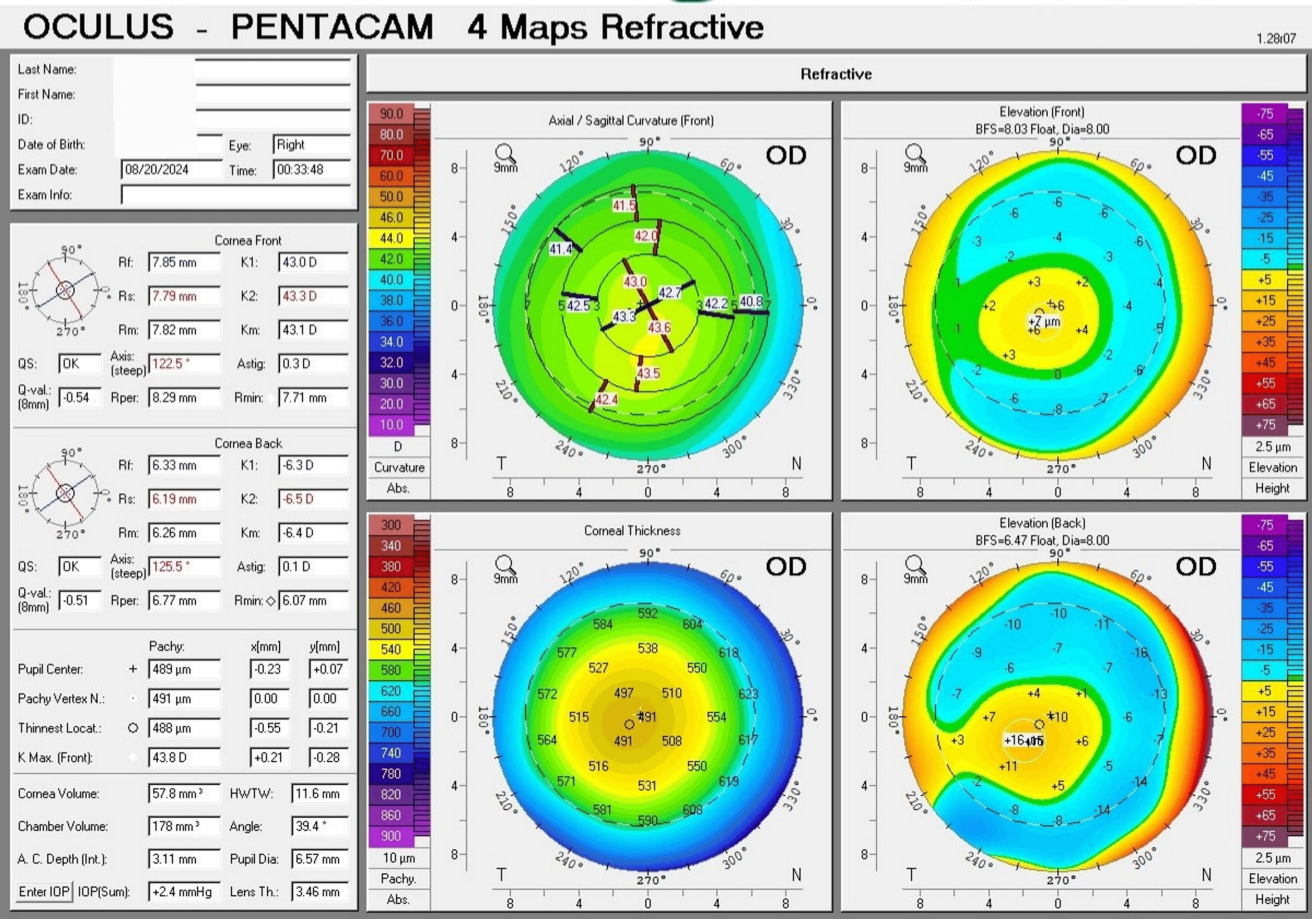
The four refractive maps of corneal tomography (Pentacam) for the right eye exhibit minimal changes and indications of subclinical keratoconus. The curvature map shows asymmetric curvature and slightly abnormal localized steepening. The thickness map indicates a thickness at the center of less than 500 µm. The front and back elevation maps are within normal limits.

**Figure 2:**
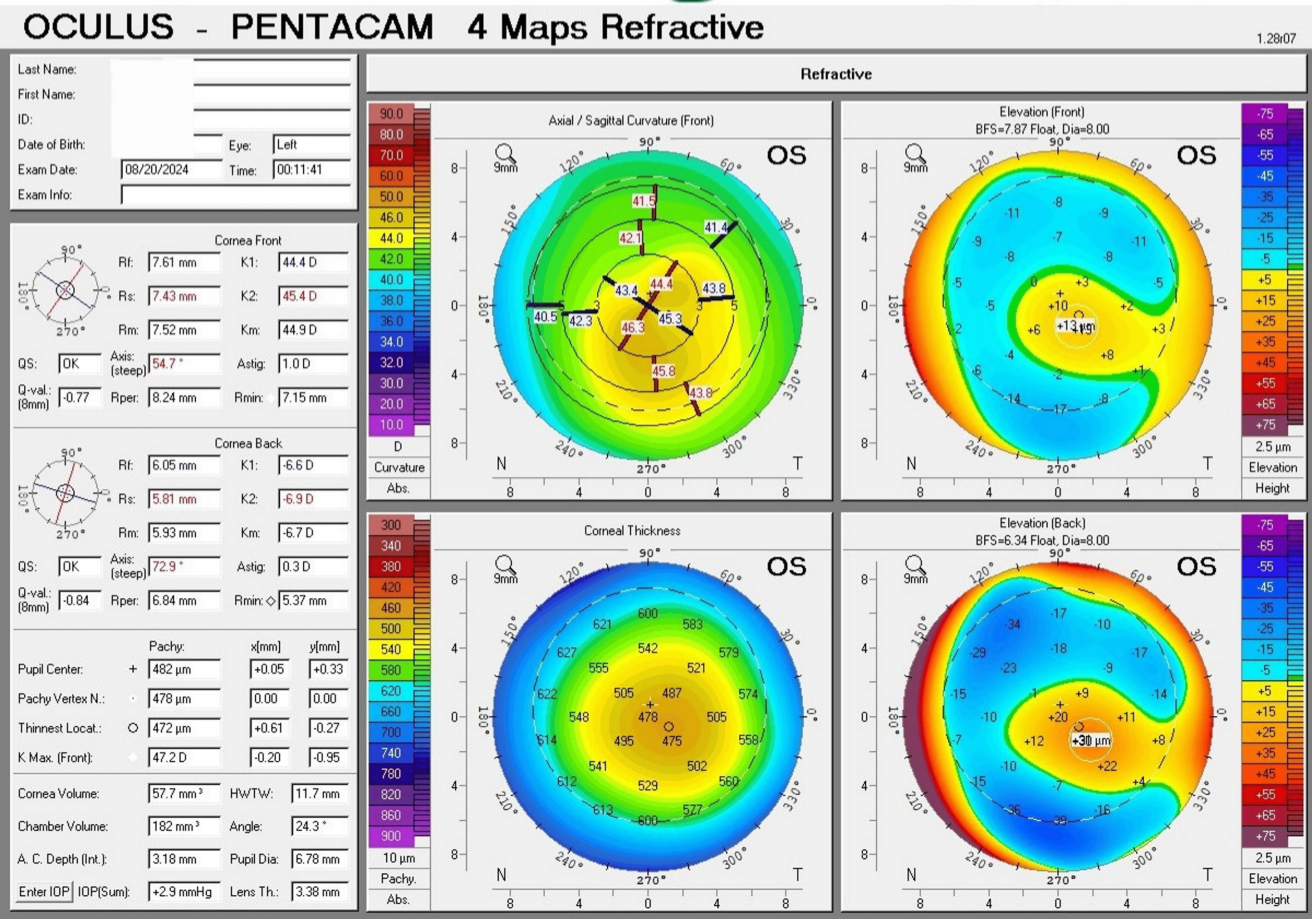
The four refractive maps of corneal tomography (Pentacam) for the left eye exhibit a pattern of mild (Grade 1) keratoconus. The curvature map shows an early oval cone. The thickness map indicates a thickness at the center of less than 500 µm. The elevation maps show suspicious elevation for the front surface and abnormal elevation for the back surface.

The four refractive maps of corneal tomography (Pentacam) showed several important parameters for keratoconus diagnosis. For the right eye, K1 = 43.0 D (7.85 mm), K2 = 43.3 D (7.79 mm), Km = 43.1 D (7.82 mm), Kmax (front) = 43.8 D, and an astigmatism of 0.3 D. The thinnest location of the cornea was 488 µm, and the corneal thickness at the pupil center was 489 µm. The highest point for front elevation was +7 µm, and for back elevation, it was +16 µm (Figure 1). For the left eye, K1 = 44.4 D (7.61 mm), K2 = 45.4 D (7.43 mm), Km = 44.9 D (7.52 mm), Kmax (front) = 47.2 D, and there was an astigmatism of 1.00 D. The thinnest location of the cornea was 472 µm, and the corneal thickness at the pupil center was 482 µm. The highest point for front elevation was +13 µm, and for back elevation, it was +30 µm (Figure 2).

**Figure 3:**
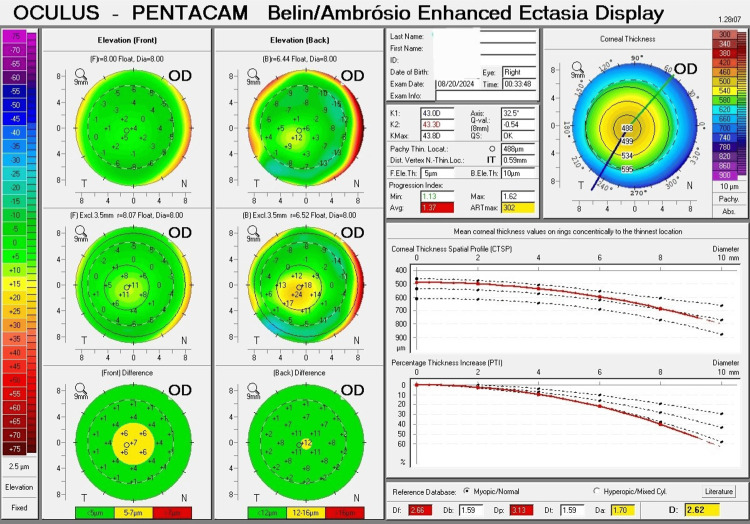
The Pentacam Belin and Ambrósio Display (BAD) for the right eye exhibits a pattern of subclinical keratoconus. The average pachymetric progression index (PPI-Avg) shows an abnormal value. The final D value and maximum Ambrósio's relational thickness (ART-max) indicate suspicious values.

**Figure 4:**
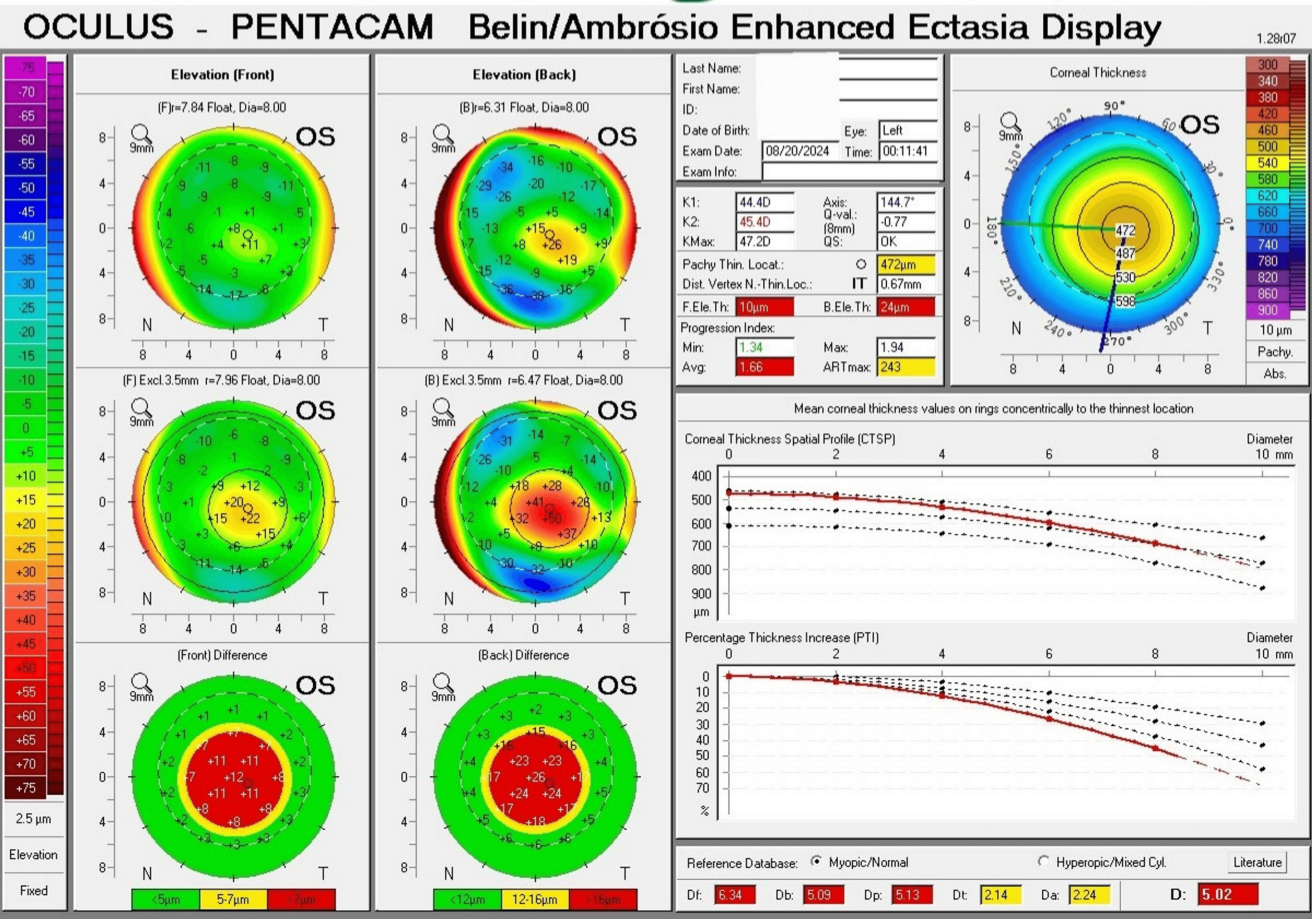
The Pentacam Belin and Ambrósio Display (BAD) for the left eye exhibits a pattern of mild stage (Grade 1) keratoconus. The average pachymetric progression index (PPI-Avg) and final D value indicate abnormal values. The maximum Ambrósio's relational thickness (ART-max) shows a suspicious value.

The Pentacam Belin and Ambrósio Display (BAD) showed several important parameters for keratoconus diagnosis. For the right eye, the average pachymetric progression index (PPI-Avg) was 1.37 (red, abnormal). The final D value was 2.62 (yellow, suspicious). The maximum Ambrósio's relational thickness (ART-max) was 302 (yellow, suspicious) (Figure 3). For the left eye, the average pachymetric progression index (PPI-Avg) was 1.66 (red, abnormal). The final D value was 5.02 (red, abnormal). The maximum Ambrósio's relational thickness (ART-max) was 243 (yellow, suspicious) (Figure 4).

After confirming the diagnosis of keratoconus, the patient was prepared to undergo corneal collagen cross-linking (CXL) for the left eye only. Following the successful surgical intervention (CXL) and postoperative follow-up in the corneal specialty clinic, the patient was referred to the optometry clinic for reassessment.

Glasses were prescribed for the patient with anti-reflective (AR) coating: −0.25/−0.50 x 180 for the right eye and −0.25/−1.00 x 180 for the left eye. Visual acuity with glasses was 20/20 in both eyes. Finally, we advised the patient to follow up every three to six months to monitor her condition.

## Discussion

This case report provides a practical explanation and clinical evidence supporting the effectiveness and reliability of the retinoscope in the early detection of keratoconus. In this study, we considered corneal tomography (Pentacam) to be the gold standard test for diagnosing keratoconus and employed the Amsler-Krumeich classification to classify the condition.

The patient's refraction and unaided visual acuity for both eyes were close to normal limits, and this data aligns with the subclinical and early stages of keratoconus. Due to subtle changes in the retinoscopic reflex in the initial stages of keratoconus, less experienced optometrists may have difficulty detecting this condition.

It's important to distinguish between the primary detection of keratoconus and its confirmed final diagnosis. In primary eye care, early detection of keratoconus is essential, even if a definitive diagnosis isn't immediate. Referring the patient to a corneal specialty clinic is necessary for final confirmation [7].

Goebels et al. compared retinoscopy, Pentacam, and the Ocular Response Analyzer for diagnosing keratoconus. While the three tools displayed inconsistent results in staging keratoconus, retinoscopy achieved better overall results, with a sensitivity and specificity of 94% and 80%, compared to the Pentacam, and 84.4% and 79.1% compared to the Ocular Response Analyzer [8]. Retinoscopy accurately detected keratoconus in 98% of cases and correctly identified non-keratoconus cases in 78%, as opposed to Pentacam's Belin and Ambrósio display, making it a highly dependable and precise test, even for the early stages of the condition [6].

While an ideal diagnostic test would have 100% sensitivity and specificity, this is often infeasible in medicine. Tests with sensitivity and specificity exceeding 90% are generally regarded as acceptable [9].

Future studies could evaluate the keratoconus rate by employing a retinoscope as a screening tool and verifying suspected cases with corneal tomography in eyes displaying an abnormal or doubtful retinoscopic reflex. This approach would allow for screening a larger and more regionally varied population, lowering the number of individuals needing tomographic corneal imaging [6].

In my clinical experience, the retinoscopic reflex pattern in the initial stages of keratoconus closely mimics the pattern observed in conditions where epithelial defects are present, such as dry eye and keratitis. This similarity arises from the fact that, in the initial stages of keratoconus, histopathological alterations mainly affect the anterior corneal layers, including the epithelium, Bowman's layer, and stroma [1].

Tomography (Pentacam) Belin/Ambrósio Enhanced Ectasia Display (BAD) is a quick and effective screening test for subclinical and early stage keratoconus. However, this device is expensive and unavailable in rural and impoverished areas, similar to the retinoscope.

Glasses with an anti-reflective (AR) coating were prescribed for the patient to work in conjunction with her prescription lenses to safely and effectively reduce glare. AR-coated glasses allow more light to transmit through the lenses while minimizing the distraction of unwanted halos and glare.

This study is restricted by its retrospective design. Additionally, the singular nature of this case study could obstruct the ability to draw universal conclusions from its findings. We advised the patient to schedule follow-up visits every three to six months to monitor the development of corneal steepening, thinning, and subsequent visual changes. These follow-ups also allow for prescription adjustments if needed. "After examining the patient and providing a final diagnosis and management plan, she expressed her deep gratitude for our care and the quality of our service."

## Conclusions

This case report highlights the importance of retinoscopy in the early detection of keratoconus. This study demonstrates the clinical significance of retinoscopic reflex patterns in the initial stages of keratoconus for the early detection of the condition. Due to its simplicity, portability, low cost, and ease of use, the retinoscope is particularly well-suited for the early detection of keratoconus and is especially beneficial in resource-limited settings.
